# 16p11.2 Microduplication Syndrome with Increased Fluid in the Cisterna: Coincidence or Phenotype Extension?

**DOI:** 10.3390/genes14081583

**Published:** 2023-08-03

**Authors:** Lívia Polisseni Cotta Nascimento, Rafaella Mergener, Marcela Rodrigues Nunes, Victória Feitosa Muniz, Juliana Rossi Catao, Ana Kalise Böttcher da Silveira, Luiza Emy Dorfman, Carla Graziadio, Paulo Ricardo Gazzola Zen

**Affiliations:** 1 Graduate Program in Pathology, Federal University of Health Sciences of Porto Alegre (UFCSPA), Porto Alegre 90050-170, RS, Brazil; dralivia.genetica@gmail.com (L.P.C.N.); rafa.mergener@gmail.com (R.M.); victoriafmuniz@gmail.com (V.F.M.); 2 Medical Residency Committee, Federal University of Health Sciences of Porto Alegre (UFCSPA)/Brotherhood of the Santa Casa de Misericórdia of Porto Alegre (ISCMPA), Porto Alegre 90050-170, RS, Brazil; marcela.r.nunes@gmail.com; 3 Medicine Course, Federal University of Health Sciences of Porto Alegre (UFCSPA), Porto Alegre 90050-170, RS, Brazil; julianacat@ufcspa.edu.br; 4 Undergraduate Program in Biomedical Science, Federal University of Health Sciences of Porto Alegre (UFCSPA), Porto Alegre 90050-170, RS, Brazil; ana.silveira@ufcspa.edu.br; 5 Health School, University of Vale do Rio dos Sinos (Unisinos), São Leopoldo 93022-750, RS, Brazil; luizadorf@gmail.com; 6 Department of Clinical Medicine, Federal University of Health Sciences of Porto Alegre (UFCSPA)/Brotherhood of the Santa Casa de Misericórdia of Porto Alegre (ISCMPA), Porto Alegre 90050-170, RS, Brazil; carlagraziadio@uol.com.br

**Keywords:** 16p11.2 microduplication syndrome, Blake’s pouch cyst, Mega cisterna magna

## Abstract

We report the first case of a child with 16p11.2 microduplication syndrome with increased fluid in the cisterna magna seen on magnetic resonance imaging (MRI). This finding may correspond to a Blake’s Pouch Cyst (BPC) or a Mega Cisterna Magna (MCM), being impossible to differentiate through image examination. The molecular duplication was diagnosed using chromosomal microarray analysis with single nucleotide polymorphism (SNP). We review the clinical and neuroimaging features in published case reports in order to observe the findings described in the literature so far and present a skull three-dimensional model to contribute to a better understanding. Despite the variable expressivity of the syndrome being well known, there is no case described in the available literature that mentions the association of 16p11.2 microduplication and the presence of BPC or MCM seen in neuroimaging exams. This finding may represent an extension of the phenotype not yet reported or may present itself as a coincidence in a child with various malformations.

## 1. Introduction

Chromosome 16p11.2 duplication syndrome (OMIM #614671) [1] is a genetic condition caused by extra genetic material due to a duplication in the region 11.2 of chromosome 16 short (p) arm. It is a very rare condition, according to Orphanet (Orpha: 370079) [2]. The prevalence is less than 1 case for 1,000,000 individuals, also reported at rates of 0.035% and 0.053% in the general population [3]. This microduplication, as well as its reciprocal microdeletion syndrome (OMIM #611913; Orpha: 261197), are supposed to arise due to non-allelic homologous recombination (NAHR) between segmental duplications, in which the short arm of chromosome 16 is rich, and predisposes to misalignment in this area [4]. Furthermore, around 70% of 16p11.2 duplications have a family inheritance [5,6,7].

Chromosome 16p11.2 duplication syndrome is a rare disease that encompasses a group whose individual prevalence is low, but as a whole, there are more than six thousand, mostly genetic (80%), which affect up to 10% of the population. The concept of rare diseases, as given by the World Health Organization (WHO), defines them as a clinical condition that affects 65:100,000 individuals, which is equivalent to 1:1538. A variety of diseases are considered rare, whose treatment is quite diverse and often nonspecific, but which have high morbidity and mortality due to their chronicity and associated complications. In Brazil, an ultra-rare disease is considered to be a chronic, debilitating or life-threatening disease with an incidence of less than or equal to one case per every fifty thousand inhabitants. The rarity of the syndrome’s occurrence makes it difficult to obtain case series that would allow a detailed description of its characteristics, making the description of case reports essential for its understanding.

Despite its rarity, several case reports are described in the literature, which allows a better clinical understanding and prognosis of the patient with this duplication. However, there is no consensus on the clinical features criteria for this comorbidity. The variable expressivity and incomplete penetrance of chromosome 16p11.2 microduplication make it challenging to suspect the diagnosis clinically. As a result, carriers of the genetic change may have no unusual clinical features, while others may have minor or even severe anomalies, leading to a broad phenotypic spectrum.

There is a wide variety of dysmorphias described such as microcephaly, triangular face, deep-set eyes, upslanting or narrow palpebral features, broad and prominent nasal bridge, finger/hand anomalies and short stature [2]. In addition, neurological and behavioral changes have already been described associated with this rare condition. Developmental or psychomotor delay, particularly of speech, motor coordination difficulties, intellectual disability, susceptibility to mental health problems, including attention deficit hyperactivity disorder, autism spectrum disorder and behavioral problems, [2,8,9,10,11] and vulnerability to seizures was also described [10,11,12,13].

The cognitive symptoms can be a result of alterations in brain physiology and/or morphology. Some copy number variations (CNVs) were already associated with changes in morphology as a partial deletion of the long arm of chromosome 3 associated with Dandy–Walker Malformation and a deletion of chromosome 11 associated with Blake’s pouch cyst, for example [14]. Mega cisterna magna was also seen in a patient with Prader–Willi (del 15q11.2–14) and trisomy of chromosome 18 [15,16].

The Dandy–Walker is a severe malformation that encompasses vermian hypoplasia with cephalad rotation of the vermian remnant, cystic dilatation of the posterior fossa communicating with the fourth ventricle and the enlargement of the posterior fossa causing an abnormally high tentorium and torcular, the latter lying above the level of the lambdoid. There is also a Dandy–Walker variant, where the vermis is present and the posterior fossa is not as enlarged as in the classic malformation. However, the demarcation between the classical and the variant is vague, and thus the term Dandy–Walker continuum is more appropriate [15].

Both Blake’s pouch cyst (BPC) and Mega Cisterna Magna (MCM) have been included in the “Dandy–Walker continuum”. The term MCM has been applied to a large retrocerebellar cerebrospinal fluid (CSF) which freely communicates with the fourth ventricle making the measure of this structure, that normally is 3-8 mm, exceeds 10 mm [15]. On that, the vermis and cerebellar hemisphere are normal. On the other hand, BPC is a rare posterior fossa cystic lesion characterized by the posterior ballooning of the superior medullary velum into the cisterna magna [17]. The differences between those are impossible to be established through just an image examination.

Here, we report the first case of a dysmorphic child with 16p11.2 microduplication syndrome, diagnosed by a single-nucleotide polymorphism-based chromosomal microarray analysis (SNP array), with increased fluid in the cisterna magna seen on magnetic resonance imaging (MRI) which may be compatible with mega cisterna magna or Blake’s pouch cyst.

## 2. Case Description

A newborn male, 30 h old at the first exam, was admitted to the neonatal intensive care unit (NICU) of the Hospital da Criança Santo Antônio, at Irmandade da Santa Casa de Misericórdia de Porto Alegre (ISCMPA), South of Brazil, due to heart disease observed via fetal echocardiography. The child was born by cesarean delivery, at term, with no complications. He had a birth weight of 2.224 kg, a length of 40 cm, a head circumference of 30.5 cm, below the third percentile on the growth curve for all values, and an Apgar score of 9/9. He is the first child of a young, non-consanguineous couple from a lower-economic-class family. His mother had adequate prenatal care and denied using alcohol, tobacco, drugs and medication during pregnancy. A fetal echocardiogram showed aortic arch hypoplasia and severe aortic coarctation. Short bones, below the third percentile, were identified during the fetal morphology ultrasound.

In the dysmorphological examination of the newborn, we observe a bulbous nose, overfolded helix, low-set backward rotated ears, the impression of a short neck, bilateral cryptorchidism, discreet clinodactyly of the fifth finger and somewhat prominent calcaneus. In addition, a single umbilical artery was observed. The agenesis of the corpus callosum was seen by cranial ultrasound. Abdominal ultrasound, high-resolution karyotype and fluorescence in situ hybridization (FISH) analysis for 22q11.2 microdeletion were normal.

**Figure 1:**
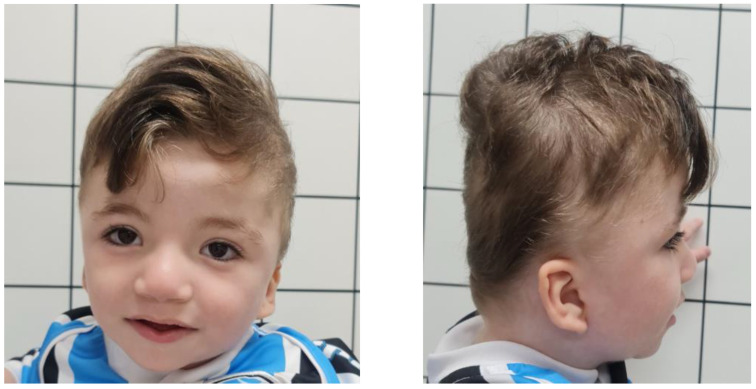
Patient’s dysmorphism at 1 year and 8 months old: brachycephaly, bitemporal narrowing, epicanthal folds, down slanting palpebral fissures and hypertelorism. Shallow nasal bridge, bulbous nose tip, low implantation of ears, overfolded helix and webbed neck.

At 9 months old, the child underwent aortoplasty and was hemodynamically stable. Dysmorphology examination showed important brachycephaly, bitemporal narrowing, epicanthal folds, down-slanting palpebral fissures, hypertelorism, a shallow nasal bridge, a bulbous tip of the nose, low implantation of ears, an overfolded helix and a webbed neck (Figure 1). Rectus abdominis diastasis and bilateral cryptorchidism were also observed. On the limbs, a single flexion crease on the fifth finger bilaterally, hypoplastic nails and the overlapping of the second and fourth toes on the third bilaterally was observed. At this time, the child presented an important delay in neuropsychomotor development. He had no cephalic support, absent voluntary palmar pressure and could not sit or crawl yet.

At 1 year and 8 months old, the child was being followed up by a physiotherapist, speech therapist and occupational therapist three times a week. Despite all the complementary therapies, the child maintained a developmental delay with muscle hypotonia, significant motor development delay, social interaction difficulties and microcephaly. He also attended the services of otorhinolaryngology, pediatric cardiology, pediatric orthopedics, endocrinology, gastropediatrics, pediatric surgery and neuropediatrics, in addition to genetics. The child evolved with severe dysphagia for all consistencies resulting in malnutrition and failure to thrive, remaining below the growth curve in height, weight and head circumference. Audiological examinations with brainstem auditory evoked potential testing showed the presence of a conductive component at various frequencies and a slight lowering at high frequencies.

The videofluoroscopy of swallowing demonstrated severe oropharyngeal dysphagia. Brain MRI confirmed the absence of the corpus callosum, and a cystic malformation of the posterior fossa, which admits the differential diagnosis with mega cisterna magna and/or Blake’s pouch cyst. In addition, the MRI showed a dysmorphism of the cranial vault, with a disproportion of the craniocaudal diameter (Figure 1). On the Doppler echocardiogram, despite the morphological alteration, the mitral valve function was normal and the postoperative coarctation of the aorta had adequate results.

**Figure 2:**
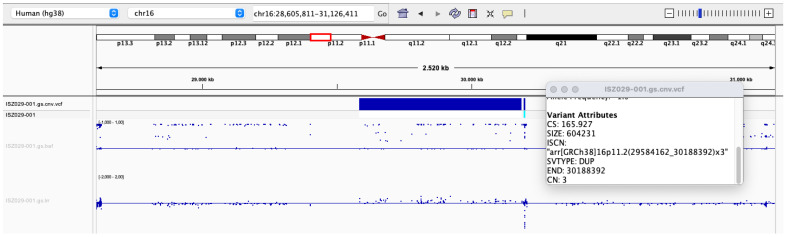
SNP array analysis in humans (hg38). Data from GSA of chromosome 16 showing the 604.231 base pairs (29584162_30188392) on the 11.2 band of its short arm.

An SNP array analysis was performed via a Global Screening Array (GSA) v3 (Illumina Technology). The SNP array found a duplication of 604 kb on the short arm of chromosome 16—arr[GRCh38/hg38] 16p11.2 (29584162_30188392)x3—compatible with 16p11.2 Microduplication Syndrome (Figure 2). This region encompasses a total of 28 genes, being 27 (*SPN*, *QPRT*, *C16orf54*, *ZG16*, *KIF22*, *MAZ*, *PRRT2*, *PAGR1*, *MVP*, *CDIPT*, *SEZ6L2*, *ASPHD1*, *KCTD13*, *TMEM219*, *TAOK2*, *HIRIP3*, *INO80E*, *DOC2A*, *C16orf92*, *TLCD3B*, *LOC112694756*, *ALDOA*, *PPP4C*, *TBX6*, *YPEL3*, *GDPD3*, *MAPK3*) completed duplicated and one (*CORO1A*) disrupted. For the child, the test was possible only by judicial request, with funding from the State of Rio Grande do Sul, as it is an exam not foreseen by the Brazilian national health system, Sistema Único de Saúde (SUS), and the family could not afford it. Unfortunately, it was not possible to carry out a molecular test for the proband’s parents as they are not eligible to have this test paid for by the Brazilian government.

**Figure 3:**
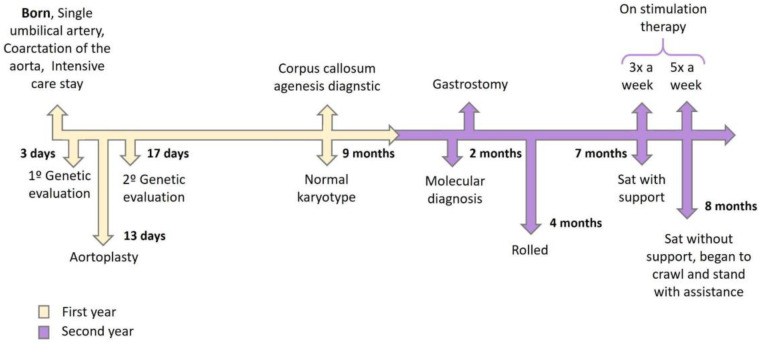
Patient’s timeline from delivery to the last follow up. The yellow arrows indicate the events from the first year of patients’ lives while the purple arrows refer to his second year.

At 2 years of age, the child underwent gastrostomy due to severe malnutrition. He awaits orchiopexy and maintains medical follow-up and complementary therapies. Relatives reported only one seizure episode associated with a high fever at the time. At 2 years and 8 months, with 20 h of stimulation therapies per week (five times a week), the child is able to sit without support (only on the ground), starts crawling and stands up with support. However, he still does not respond to sound stimuli, does not speak (absent speech) and does not swallow food. The timeline with the exams, surgical procedures performed and development milestones can be followed in Figure 3.

So far, cognitive and motor development delays are known, but it is not possible to determine other neuropsychiatric and behavioral characteristics commonly associated with the diagnosis due to young age. The WISC test for IQ assessment has not been performed yet. Other cognitive assessment scales for ages under 3 years old are not available in the country’s public health system and have not been carried out.

**Figure 4:**
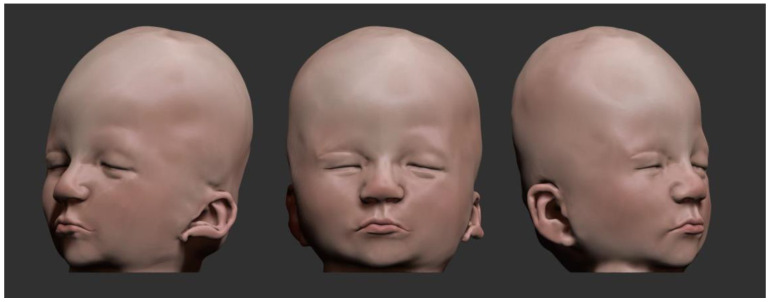
Three-dimensional illustration highlighting cranial morphology and dysmorphias. The low implantation of the ears and brachycephaly can be easily visualized.

**Figure 5:**
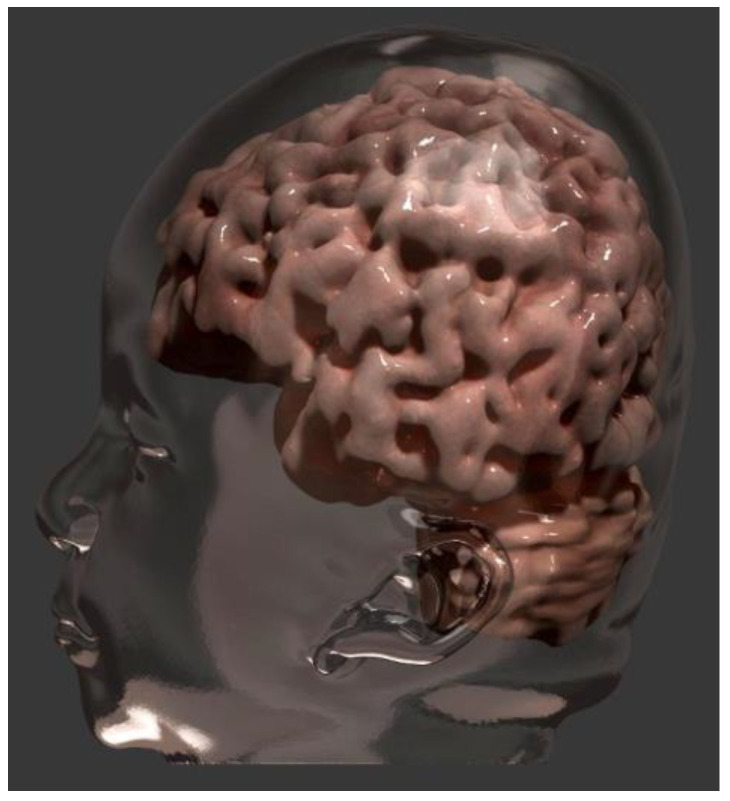
Three-dimensional illustration highlighting the head (transparent) and encephalic morphology. It is possible to notice the brachycephaly causing flattening of the occipital lobe.

Genetic counseling was offered to the family, and the outpatient genetics service will continue to monitor the child’s development. To portray the patient accurately, we developed a three-dimensional model of cranial morphology and dysmorphias (Figure 4 and Figure 5). The patient’s parents agreed to participate in the study and informed consent was given and signed afterwards. The study was approved by both institutional Ethics Committees.

**Figure 6:**
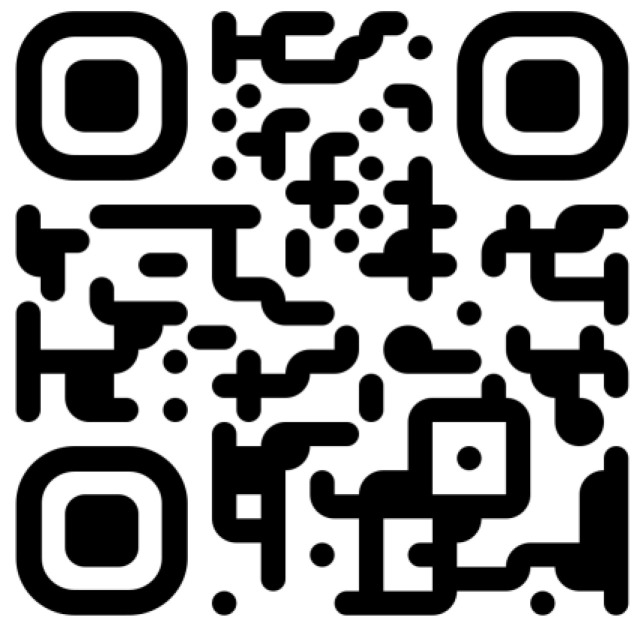
QR code to access the 3D model.

As mentioned, by utilizing advanced computer graphics techniques, we performed a three-dimensional reconstruction of the patient’s skull from MRI using the *3dSlicer* software. Subsequently, we further enhanced the model in *Blender*, applying refinements, painting and rendering to achieve an even more realistic visual representation (Figure 5). This refinement stage allowed the addition of precise details to the three-dimensional model, ensuring a faithful representation of the dysmorphias present in the patient. The online availability of the model extends its reach and increases the accessibility of scientific content, providing an accessible and didactic means of visualizing and studying dysmorphias. The three-dimensional model resulting from this work is available for free viewing through the QR code (Figure 6).

## 3. Discussion

It is well known that the chromosome 16p11.2 duplication presents variable expressivity, with clinical features ranging from subclinical to severe. However, there is no case described in the available literature that mentions the association of this syndrome with increased fluid in the cisterna magna seen on MRI. For this reason, we would like to raise the discussion about a possible extension of the phenotype and the importance of researching neuroimaging of patients with 16p11.2.

Considering the high percentage of familial inheritance with asymptomatic parents, it is essential to agree on the importance of the microarray for the patient’s parents. Unfortunately, it was not possible to carry out an SNP array for the parents. They could not afford the test. As they do not have clinical manifestations, they are not eligible to have this test paid for by the Brazilian government, and a judicial request was rejected.

Recent articles address the frequent association between 16p11.2 microduplication and neuropsychiatric disorders, such as autism spectrum disorder (ASD) [7,18,19] schizophrenia [9,18] and other neurodevelopmental disorders including developmental delay, intellectual disability, attention deficit hyperactivity disorder (ADHD) [6,7] developmental coordination disorder and language disorders [20,21]. The child in the present case already presents a global developmental delay (GDD), absent speech and feeding difficulties due to hypotonia, associated with difficulty in social interaction.

Furthermore, he has not yet shown signs or symptoms of neuropsychological disorders, but we will continue to follow up in the next few years conscious of the fact they may appear. The WISC test for IQ assessment has not been performed yet, and other cognitive assessment scales are not available in the public health system so those have not been carried out so far. The diagnosis of these neuropsychological disorders may be difficult due to a phenomenon known across genetic disorders as “shift”. A shift may happen when a child has an average IQ score compared with the general population; however, compared to their above-average parents’ score, this could represent a significant deviation from the expected outcome (i.e., an IQ score similar to that of the parents). Thus, when examined out of family context, their average cognition gives the illusion of nonpenetrance. Depending on the familial starting point for a certain trait, a shift could cause an individual to reach or not reach the diagnostic parameters for a clinical neuropsychological disorder [22].

GDD is defined as a significant delay in two or more domains of development in children younger than five years old [23,24]. The present report shows a child with GDD who achieves significant improvement in the development milestones after starting stimulation therapies. It is important to pay attention to the child’s neurodevelopment since one of the most common causes of delay in prenatal development is genetic diseases [23]. Children need to be referred for stimulation therapies as early referral and proper management positively alter the child’s trajectory [23]. It was proposed that conventional rehabilitation associated with multisensory stimulation therapy can better help in the development of cognition and movement, improving the quality of life and reducing the disability of these children [24].

Recently, differences in brain structure and some significant MRI findings of people with 16p11.2 microduplication syndrome were described. Deletions and duplications of the 16p11.2 locus have already been associated with changes in brain anatomy [25,26]. Within those diagnosed with a duplication, microcephaly, cerebral white matter or corpus callosum abnormalities and ventricular enlargement were also observed, but less frequently than speech articulation abnormalities, hypotonia, abnormal agility, sacral dimples and seizures or epilepsy [26,27].

Regarding the central nervous system, 22.3% of duplication carriers were reported with microcephaly [3]. Other features include prominent cisterna magna and sparse gyration [28], expanding the range of cephalic phenotypes associated. It has also been described, in magnetic resonance studies, as reciprocal changes in brain volume with a decrease in gray/white matter. Several brain areas such as the insula, calcarine cortex, accumbens, pallidum, transverse temporal gyrus, caudate, putamen and thalamus exhibit reduced volume [3]. The patients also show decreased axial diffusivity of white matter and thinning of the corpus callosum. It was also found that human-induced pluripotent stem cell-derived neurons from 16p11.2 duplication carriers display corresponding features with reduced size/dendrite length in neurons [3]. Furthermore, the reduced cortical surface area has been reported, and analyses of cortex anatomies revealed reduced cortical thickness in both 16p11.2 deletion and duplication carriers [26].

In order to elucidate the complexity of these findings, we selected case reports and case series that provided a review of the cranial and neuroimaging features. Additionally, we create a table with the findings described in the literature (Table 1). Microcephaly was the most common cranial feature, observed in 8 out of 15 patients (53.33%). Craniosynostosis was observed in only one patient, but most of the patients underwent only MRI analysis and did not have computerized tomography scans (CT). Brachycephaly was only described in the present case, and one patient was described with a flat occiput. Although flat occiput can be present in patients with brachycephaly, it is considered a distinct finding [29].

Agenesis of the corpus callosum is observed only in two patients, including the present case (13.33%). Other abnormalities of the corpus callosum such as thinning and dysplasia are quite common and have been described in six patients (40%). Another common abnormality is the presence of white matter hyperintensities, also observed in six patients (40%), followed by ventriculomegaly, present in four reports (26.6%) and cortical atrophy present in three reports (20%). Additionally, three of the reports compared in the table included patients with normal MRI findings.

However, the most intriguing finding in our case was the presence of a well-formed cerebellar vermis, with only a liquid compensation cyst being observed. Therefore, it does not meet the criteria for Dandy–Walker malformation, defined by the triad: cystic dilation of the fourth ventricle, the hypoplasia and upward rotation of the cerebellar vermis and an enlarged posterior fossa with the upward displacement of the lateral sinuses, tentorium and torcular [30,31]. The referred liquid compensation cyst makes a differential diagnosis with BPC and MCM, making it impossible to differentiate them via image analysis. Radiographically, BPC and MCM are seen as cystic masses in the posterior fossa [32] and both present with an intact cerebellum.

According to embryology, it has been mentioned that the persistence of the pouch with variable separation of the fourth ventricle and lack of communication with the subarachnoid space results in enlargement and BPC [28]. When the vermis, cerebellar hemispheres and fourth ventricle are normal, an area of posterior membranous defect can produce MCM and/or persistent BPC as two distinct malformations [33,34].

BPC is considered one of the anomalies within the spectrum of the Dandy–Walker complex by many authors [34]. Although, there are authors who support the opinion that it does not fit into the Dandy–Walker spectrum, as the fourth ventricle does not communicate with the subarachnoid spaces in the midline [28], maintaining the controversy. A persistent Blake’s pouch cyst forms when proper permeable organization to constitute the foramen of Magendie does not occur [28,32]. The radiological characteristics are: tetraventricular hydrocephalus, infra or retrocerebellar location, presenting well-developed and non-rotated cerebellar vermis, the cystic dilation of the fourth ventricle without cisternal communication and some degree of compression in the medial cerebellar hemispheres [34]. However, they are not associated with other brain abnormalities [32].

On the other hand, MCM is now considered a congenital cystic malformation of the central nervous system characterized by an enlarged cisterna magna, the absence of hydrocephalus and preserved cerebellar vermis [32]. It is believed that its embryological origin is from the permeability of BPC [32]. Although this condition is generally asymptomatic, it has been associated with a number of neurological symptoms, including headaches, vertigo, ataxia and developmental delays, as well as psychiatric conditions (such as mania, autism, catatonic schizophrenia and mild syndromic intellectual disability), with impaired memory and verbal fluency [32,35]. The underlying genetic mechanisms involved in the development of MCM still need to be fully understood. However, studies have suggested a possible genetic link between mega cisterna magna and various chromosomal abnormalities, including microdeletions and microduplications of specific genomic regions [32,36].

It has been proposed that the inclusion of molecular and genetic analysis could be of great importance for the definitions of Dandy–Walker and Blake’s cyst as a variant and/or continuum [34]. This could allow a future initiative to search for molecular alterations that may correlate with BPC or MCM, in order to help in the differentiation after radiological examinations. After all, conventional imaging analyzes are not sensitive enough to detect obstructive membranes in the cerebrospinal fluid [37]. Descriptions as we are proposing here can shed light on genes or pathways involved, but for now, it still remains inexistent.

**Table 1:** Review of the clinical and neuroimaging features described in the literature. Cranial features and neuroimaging abnormalities (MRI/CT scans) of patients with duplications in the chromosomal region 16p11.2 compared to the ones observed in our patient.

	Present Case	Behjati et al., 2008 [38]	Bedoyan et al., 2010 [39]	Tabet et al., 2012 [40]		Filges et al., 2014 [41]			Dastan et al., 2016 [42]	Shim et al., 2019 [43]	Posar et al., 2020 [20]		Lengyel et al., 2020 [4]	Levkova et al., 2021 [44]	Butler et al., 2022 [45]	Frequency
Patient				P2	P3	P1	P2	P3			P1	P3	P5		P1	
Microcephaly	+	+	+				+		+	+				+	+	53.3%
Brachycephaly	+															6.66%
Flat occiput	+								+							13.3%
Craniosynostosis														+		6.66%
Other abnormalities of the corpus callosum		+	+	+	+		+				+					40%
Agenesis of the corpus callosum	+														+	13.3%
Ventriculomegaly						+	+					+	+			26.6%
Cystic malformation of the posterior fossa (mega cisterna magna or Blake’s pouch cyst)	+															6.66%
Cortical atrophy				+	+		+									20%
White matter hyperintensities				+	+	+	+	+					+			40%

MRI: magnetic resonance imaging; CT: computed tomography; +: present.

Although several neuroimaginological changes have already been described, there is no description in the current literature of increased fluid in the cisterna magna as it appears in the present case. Moreover, there are only a few patients with 16p11.2 microdeletion published in reports that mention the existence of an MRI and address its results in detail. We believe it would be important that asymptomatic carriers of the duplication be evaluated via MRI in order to observe whether a lower amount or an absence of fluid in the posterior fossa is related to the less evident neuropsychiatric phenotype. Additionally, it would be of great value to have new reports of patients with 16p11.2 microduplication to consider neuroimaging as part of the evaluation in order to better understand the correlation of the increased fluid in the cisterna with that genetic condition.

It is important to consider performing another molecular test, as an exome sequencing analysis, considering the patient’s extensive dysmorphisms to evaluate additional diagnoses. Analysis via exome sequencing is also not foreseen in the Brazilian national health system, SUS. Since it is a high-cost test that would not change the management and survival of the patient, it was not authorized by the court by judicial decision. Unfortunately, nowadays, developing countries find costs as a significant barrier to scientific research, especially regarding rare diseases.

## 4. Conclusions

In conclusion, the presented report raises the hypothesis of a possible correlation between 16p11.2 microduplication and posterior fossa malformation. This report is the first to document the presence of increased fluid in the cisterna magna in a patient with 16p11.2 duplication, compatible with mega cisterna magna or Blake’s pouch cyst. This finding may represent an extension of the previously unreported 16p11.2 duplication-associated phenotype, or it may prove to be a concomitant but isolated event in a child with multiple malformations.

Therefore, we highlight the need to investigate posterior fossa abnormalities through magnetic resonance imaging in other patients with the same microduplication. Due to the incomplete penetrance and variable expressivity of this condition, it is crucial to explore this possible correlation in future studies to build a better understanding of the range of phenotypes linked to 16p11.2 duplication. Accordingly, new publications are still needed, either to confirm the association suggested as part of the syndrome or to identify it as a mere isolated fact. After all, the real prevalence of this finding in 16p11.2 microduplication is not known.

Another important aspect to emphasize is the great benefit of stimulation therapies for the development and better quality of life of children with developmental delays. We hope that this case report can draw attention, in addition to the genetic condition and its phenotype, to the need for international partnerships and greater access to diagnostic tests in developing countries such as Brazil. We understand that the contribution to the scientific community would be even greater if there were more comprehensive access to technologies to offer diagnoses and phenotypic correlations.

## Data Availability

Data is unavailable due to privacy and ethical restrictions.
